# Negative Space: An Alternative Framework for Archaeoacoustics

**DOI:** 10.5334/joc.331

**Published:** 2024-01-09

**Authors:** Victoria Anh-Vy Pham, Roland Fletcher

**Affiliations:** 1 St John’s College, Department of Archaeology, University of Cambridge, GB; 2 Department of Archaeology, University of Sydney, AU

**Keywords:** Music cognition, Multi-sensory perception, Auditory perception

## Abstract

Hearing the remote past seems impossible. Archaeoacoustics is a contemporary field intent on reconstructing the evolution of early communication systems, offering the possibility of developing methodologies relating to past sound signaling and music. Through a contribution of the emerging sensory field of archaeoacoustics and an example of acoustic assessments conducted at the site of Coves del Toll, can we understand signals of the past in order to investigate human behaviour and trace its cognitive evolution? This paper explores alternative methodological and theoretical approaches to understanding prehistoric sonic behaviours in early hominids and aims to set out a framework to theoretically and philosophically approach the “sound record” of the past. The theoretical proposition of this paper integrates the musical and sound art disciplines of spectralism and sound ecology to challenge the current limitations of listening to sound.

## Introduction

‘I call architecture frozen music.’

– Goethe (1839)

Schofield (2014: 289) asks, ‘What can archaeology contribute to our understanding of music?’ whereas we should seek to invert his query. What can music contribute to our understanding of archaeoacoustics? Music’s basic operations, not the social function, can offer insight into signalling processes. Music counteracts the notion that any sound that carries primary signals or meaning travels linearly or between a single transmitter and a single receiver. When processing non-textual and complex instrumental music, four to six lines of music can be simultaneously perceived in a manner that language or verbal communication cannot convey (Tymoczko 2010: 93; Warren 1999: 116–117). There is a symbiosis between forefront material, such as the main melody or effect, and ambient or “accompaniment.” Therefore, multiple streams of complex non-text-based information can be perceived as a single image. The cognitive capacity to process rhythmic cells and a melodically evolving complex harmonic spectrum indicates the significance of observing the listening experience within spectral depth.

Prehistoric music is often recreated through a series of ethnomusicological analyses of communities, such as those in Papua New Guinea (Feld 1981), Native Americans of the Plains and Africa (Morley 2006: 95–105) as a form of comparative remodelling by focusing on its sociocultural function. Like all ethnoarchaeology, it conflicts with the dislocation of time by attempting to parallel contemporary cultural behaviours with past societies (Agorsah 1990; Fletcher 2007: 10–11; Harmon 2016; Hiscock 2007; Skibo 2009; Wylie 1985). Rather than focus on a recreation or, indeed, a reimagining of prehistoric musical practices and the potential ritual function of music, integrating basic musical principles can provide an alternative platform to understand the role and generation of sound. This application of modern musical theory onto the archaeological record does not seek to re-sound the past as a contemporary classical music reinterpretation but to enforce the significance and the profound quality of the continuity of sound perception, the methods of sound perception, and potentially the continuity of music as a signalling system. A helpful method of approaching early sound-making practices than ethnomusicological research is considering bioacoustics and studies into primate drumming and percussive signalling (Dafour, Curé & Pulin, 2015).

Instead of relying upon researchers’ visual biases of perceiving and interpreting the past, in particular assumptions about prehistoric music-making, this paper seeks to turn to current concepts within music and sound and their potential to add to the framework of archaeoacoustics. In particular, this paper discusses the Western art music movement, spectralism, that emerged in the 1970s, the advent of sound ecology, and the potential for such conceptual frameworks to apply to listening to the past.

Listening to the past has long been regarded as an impossibility. It is difficult to recreate the diversity and complexity of contemporary soundscapes, let alone attempt to reconstruct, model, and understand the significance of sounds heard during the Palaeolithic. Archaeoacoustics is a field seeking to address the theoretical and methodological challenges that arise when framing archaeological research into the sensory dimension of sound (Scarre and Lawson 2006; Morley 2006, 2013; Till 2014). Often, archaeoacoustic theory refers to the emergence of language while correlating early or proto-music-making with it as a single human behavioural pattern (Fazenda et al. 2017: 1377; d’Errico & Henshilwood 2011; d’Errico et al. 2003). Methodologically, this was reinforced by mapping the acoustics of archaeological sites to pinpoint ‘acoustically resonant points’ that sought to relate resonant points with the location of features of materiality, particularly various forms of rock art (Rezkinoff & Dauvois 1988; Fazenda et al. 2017; Reznikoff 2008, 2012) and since then, by the use of Ambisonic technologies and binaural microphones to identify resonances and acoustic signatures (Goh 2017).

However, sound persists beyond what can be seen and touched. We hear ‘within sound,’ making listening an immersive, spectral experience that must be emphasised in archaeoacoustic methodologies. That the environment forms a significant proportion of our perception of place and space is undeniable. Navigating place, or sound ecologies, through acoustic perception is significant, as is an investigation into place affecting sound-making (Primeau & Witt 2018: 875). By assessing the potential evolutionary value of sound signalling, we can begin an interpretation of prehistoric sound practices that acknowledge its variation and complexity. To extend from this challenge, this paper unpacks perspectives on sound, the limitations of our current approaches to its analysis, and its application to archaeological study. Archaeoacoustics requires consideration of the operational mechanisms of sound and its perception and, thus, its effect on culture, society, and cognition. We will consider a rigorous inspection into acoustic assessments of archaeological sites by proposing a theoretical framework that includes concepts of negative space, silence, gesture, repetition, and musical principles in a move towards a more wholistic understanding of sound, its impact, and its impact upon the evolution of sound signalling and sound practices.

Prehistoric caves and the societies that occupy such sites require an analytical approach that models acoustic patterns, structure, and networks to build a sonic image of the ambient nature of space before exploring the range of deliberate sound-making that can occur in such spaces. Applying the principles of music to prehistoric acoustic archaeology does not focus on the identification or rare finding of sound-producing objects or the artistic and symbolic function of music. Our understanding of contemporary expression often frames such analyses, but acoustical principles should instead consider the dialectic between space, experience, and sociality (Blake & Cross 2015; Blesser & Salter 2009). The necessity to filter auditory information, from ambient to directed, permits an investigation into the evolving characteristics of soundscapes over time and their subsequent impact on hominid sound-based behaviours.

### Negative Space

The primary issue is the assumption that acoustic research can be approached through linear perception (the notion that any sound signal is transmitted in one direction and only receives its source) and contextual interpretation. Often through a concentration upon the hypothetical correlation of visual material such as rock art with resonance patterns or musical practices with Palaeolithic ritualistic behaviour, the symbolic function of sound dictates interpretation by centering on meaning. In contrast to this viewpoint is the actuality that sound is an immersive, spectral continuum. Sound can be technologically examined and understood from a spectral perspective; the theoretical proposition presented in this section refers to spatial representations of sound as *negative space*.

Archaeology and, by extension, archaeoacoustics rely on the physical and the visible. This dependency upon material culture poses a theoretical challenge requiring a systematic shift from dependence on material interpretation. Archaeoacoustic studies have sought to indicate intentional manipulation of spaces for potential sonic production (Reznikoff & Dauvois 1988; Dams 1984, 1985; Till 2014). This includes the manipulation of geological formations, particularly stalagmites and stalactites (Dams 1984) and coloured markings (Reznikoff 2008; Reznikoff & Dauvois 1988). Then, visualising a space through acoustics presents a set of theoretical conundrums. There is the presumption of a linear transaction between a *seen* artefact and a possibility of past sonic behaviour. For example, a prehistoric painted red or black ochre dot *could* relate to an acoustically resonant or significant location in a cave (Reznikoff 2008). The simplified, linear transaction between object, space, and sense relies upon the cultural context of the interpreter and is, therefore, unreliable (Blesser & Salter 2009: 73-74). A mis-conceptualisation of sound as a two-dimensional experience reduces the reality of its spectral nature to a ‘*line of sight*’ as we can see with vision. Thus, moving away from framing sound signalling through the fallacy of linearity provides a more holistic perspective of how sound practices, musical or linguistic, have emerged and evolved.

*Negative space* promotes the modelling or thinking of spaces inclusive of depth and three-dimensionality, whereas archaeoacoustic research has often been dictated by the dependence upon specific material objects and loci in past sites (Navas-Reascos et al., 2023; Waller 2015) or the general acoustics of the spaces, in particular, architectural spaces (Duran et al. 2021; McBride 2014; Omar et al. 2020). Similarly, the significance of addressing an archaeological site in this manner is that it does not limit archaeoacoustic thinking to interior spaces or sites of habitation, whereas archaeoacoustic research has often been dictated by the dependence upon specific material objects and loci in past sites (Navas-Reascos et al. 2023; Waller 2015) or the general acoustics of the spaces, in particular architectural spaces (Duran et al. 2021; McBride 2014; Omar et al. 2020). A systematic move into considerations of the volume of negative spaces encourages a move from a material or sociocultural-specific interpretation of acoustic environments. The proposed terminology, *negative space*, derives from art historical analysis, which refers to a ‘space’ or the transitional region that foreground material does not occupy. Adopting it for archaeoacoustic analysis, the term negative encourages visualising a physically ‘empty’ or ‘silent’ space as one with depth. Rather than a two-dimensional visualisation of resonance, such as a depiction of sound waves or relying on spectrograms, we can consider a space in its three-dimensional form.

The purpose of our theoretical acoustical framework of *negative space* refers to and applies to *all* occupied spaces, natural or artificial. Particularly relevant is the investigation of prehistoric sonic perception and behaviour in any form or space, for example, the geological formations of a karstic cave or rock shelter. Cave sites and rock shelters have long fascinated archaeoacoustic analysis, given their natural resonances and evidence of past hominid habitation (Díaz-Andreu & Benito 2012, 2014). Although this terminology has the potential to refer to any occupied space, the primary purpose is to address the conundrum of analysing acoustic perception within prehistoric occupied spaces, which would, in due course, permit comparative studies of various spaces, such as ever-evolving karstic cave complexes, as they transform through time.

In order to analyse any space, from prehistoric to contemporary, the visualisation of the presence of sound in internal spaces must be framed as a form of *negative space*. Perceiving a space as a ‘negative’ volume reinforces the notion of the omnipresence of sound. This flexible framework can be applied to various spaces, from geological formations such as La Cova de les Teixoneres in Moià, Catalunya, to even the abstract architectural forms of Gehry’s Fondation Louis Vuitton in Paris. This flexible framework permits the networking of a space’s visual and acoustically recorded aspects regardless of the placement of specific material entities. Although there will be elements of correlative behaviour available for interpretation at prehistoric sites, reframing the sound palette of the past through the concept of *negative space* shifts the primary focus of the function of space towards an analysis of sonic depth.

*Negative space* emphasises the relationships between intentional sound-making practices and ambient noise. This paper recommends taking a holistic and directional approach to human behaviour that no longer skews a reimagining of the past as a period where sonic behaviours such as music and speech were entangled. The issue with acoustic data and interpretation within a prehistoric ritual context is that such behaviour, labelled as artistic, is assumed to be early ritual behaviour rather than observational or experimental (Blake & Cross 2015: 115; Till 2014: 293). Although it cannot be denied that the purposeful structuring of sound via language and music are significant aspects of early human behaviour, occupied spaces require analysis as sound ecologies rather than being defined by object materiality.

Framing a prehistoric understanding of sound through the concept of *negative space* encourages a shift in perspective and acoustic or sonic behaviour away from cultural function or as markers of specific cultural behaviour. Negative space terminology removes the association with anything specific, such as pigmentation or artefact assemblage, and focuses purely on space. Although material objects in the archaeological record are not omnipresent, sound is.

### Disrupting Silence: Sound as Spectrum

The term spectrum is more commonly associated with the visual perception of colour. Approaching an archaeological site from a perspective of *negative space* sets up the question of how we can approach the act of listening and the network of interacting sounds we experience within it. Spatial and temporal depth in sonic experiences allows for a concentrated technical analysis of negative spaces, soundscapes, and acoustic soundscape ecologies without sound necessarily being assigned to cultural meaning and verbal signalling. The desire for sound to be understood as a linear or binaural experience has potentially influenced several methodological approaches to acoustic mapping and testing of archaeological sites (Farina 2014; Reznikoff & Dauvois 1988; Díaz-Andrue & Benito 2012; Díaz-Andreu & Benito & Lazarich 2014). It is a spectral and immersive experience. Sonic perception is not only a transaction between the ‘sound producer’ and the ‘sound receiver’ but is a sensory platform where soundscapes can be mapped as networks of continuous interactions (Farina 2014: 4–5). Rather than skewing analysis towards the deliberate function of sound as possessing linguistic meaning, cultural coding, and symbols of complex verbal communication, acoustic analysis needs to move beyond verbally interpreted sound practices. Sound should be viewed as a temporal, multi-dimensional experience and a spectrum of diverse interactions and transmissions.

The collective imagination of the past conjures up a remote past in silence. In contrast to the overwhelming din of modern life, we tend to reduce it to silence; however, human life in the deep past was far more active and sonically complex. Sound is a spectrum with temporality. Relying on physical traces of the past remains inevitable in an archaeological investigation; however, a systematic move to understanding sound and its subsequent social exploitation may alter the currently fragmentary nature of archaeacoustics. An archaeology of sound must begin by asking, what is sound itself?

Silence plays a crucial role within the spectrum of sound on two counts. First, it highlights the depth of acoustic imagery as being beyond material or meaning-based analysis of prehistoric sound interactions, and second, that signalling systems require sonic punctuation or systematic use of “silence” to be coherently communicated. Categorising “noise” as silence within archaeoacoustic analysis diminishes the complexity of sensory perception. This paper seeks to clarify that there is no such thing as silence. Affirming that there are periods of “silence” (historical, present, or naturally occurring) denies the reality of experiencing sound. Environments and spaces are processed as audio images, including background noise. We continue to hear this silence even when we are not listening.

Silence, which encompasses ambient and background or accompaniment noise, has a structural function in signalling by separating information units such as a musical tacet or linguistic phrasing. From this metaphor, we can consider ‘silence’ as necessary breaks in intentional or forefront noise as sonic punctuation. This understanding of ‘silence’ is essential to the perception of acoustic sequences to be generated and then evolved. Silence holds a cellular function in which information can be framed and transmitted coherently, ensuring the auditory context and ambient noise filter through intentional sound-making practices.

In the 1970s, classical Western art music composers, sound artists, and theorists developed the modernist movement of spectralism. Due to the increasing integration of technology into musicological analysis and artistic process, spectralism applies to the recognition and artistic manipulation of the harmonic series over a fundamental tone or pitch. Negative spaces can carry an acoustic signature or series of acoustic resonant points where tonal fundamentals can be analysed if identified. Spectralism has primarily been applied to musical composition. In music, it is necessary that we hear a beginning and we hear an end. This is to say, we recognise the start of a piece of music and can make out its conclusion in the same way that we might with listening to Shakespearian prose. To borrow from music, if we imagine a single line of melody as a single stream of sound transmissions, we may be able to perceive two streams in the context of language. To illustrate this, imagine if two speakers were addressing you at once. Although it would be possible to discern the content of the two streams, there is likely to be a struggle for coherence. However, we are capable of simultaneously understanding up to six streams of musical transmission without loss of impact or coherence (Tymoczko 2010: 93). This may be due to the inference of meaning and specificity of content involved in linguistic transmissions that music does not require. Our ability to perceive structured sound in music as being simultaneously linear through time and vertical in a layered spectrum of sound transmissions indicates that limiting our understanding of sound signalling to a series of single transmissions reduces our presumption of how sound behaviour may have evolved. Although the ability to coherently perceive many layers of transmissions in modern humans’ developed systems of language and music is evident, it underscores a significant evolution from the “silence” of the remote past and the noisy sound ecologies from which we have developed systems to navigate. Therefore, sonic behaviours may have evolved to be heard and understood spectrally.

Archaeological practice occasionally relies on speculations relating to direct or intentional sound, for example, language and potential artefacts used in early music-making, instead of the context from which these sound-based behaviours arise. Applying spectralist thinking can aid us in identifying the degree of complexity that sound perception and soundscapes can retain. Instead of hypothesising about material culture, we can consider how layers of auditory information can be filtered by sociocultural standards and by the brain and how they can interrelate. The transmission of as many as six lines of harmonic information at any given time (Tymoczko 2010: 93) can be perceived as a single signal. Integrating spectralism into archaeoacoustics ensures that sound-based research encompasses natural resonances or ambient and background noise with intentionality. Rather than separating them into two categories, it is more beneficial to emphasise the sound as a network of interactions and that the polyphony between ambience and intentionality is essential for sensory perception.

This proposal for a holistic approach, which unites with the spatialised, multi-modal proposal of *negative space*, is not unique to the 1970s spectralist movement in contemporary Western Art music. The Medieval philosophical concept of Musica Universalis proposes that ‘proportions in the movements of celestial bodies… could be viewed as a form of music; inaudible but perfectly harmonious’ (Anonymous 2008: 26). The postulate was that such large bodies possessed an inherent sonic impression or sensation that inform our subconscious sonic experiences of our universe. This concept proposes two ideas; first, the possibility of analysing sound as a structure or series of proportions, and second, that silent or inaudible noise, in actuality, forms an essential role in the acoustic fabric of sensory perception. Rather than focusing acoustic research on the dissemination of sonic meaning or the restriction of sound as a cultural by-product, commentary and theories of classical antiquity offer a structural perspective that archaeoacoustics can adopt. There has long been an understanding that sound has a depth beyond the intentional.

Since Classical Greek theorising, a notable example of exploration between the sonic boundaries of intentionality and ambience is John Cage’s infamous “silent” work, 4’33” (1952). Cage’s utilisation of silence as his primary compositional tool (Till 2014: 295) affirms that music is gestural and capable of engaging with both intentional and ambient sound. The musical employment and intentional “use of silence” affirm that there is no silence. Music, in essence, includes forefront features or primary messaging supported by accompanying lines of sound that exist within an ambient space. Listening to any music work includes an experience of the concert hall in which the work operates as part of the sonic imagery, not just the music itself. Prehistoric music and communication systems would have been inevitably embedded into its broader acoustic environment (Truax & Barrett 2011) as much as Cage imposed the sonic depth of New York City upon his audience. The ever-present background low and high frequencies are initially not perceptible if the technical focus of analysis is upon the meaningful or symbolic content of forefront audio material but is essential for perceiving the networks between space, sonic depth, and developing signalling processes. As noted by Cage (1961: 25), ‘I believe that the use of noise, wherever we are, what we hear is mostly noise. When we ignore it, it disturbs us, and when we listen to it, we find it fascinating.’ In this line of thought, an acoustic archaeological framework requires listening to “silence.”

### Polyphonic Perception in *Negative Space*

As a counterpart to the depth and acoustic signatures embedded within a negative space framework, polyphonic perception considers sound as a spectral experience. The proposition of using the term polyphony is not without history. Through spectral studies of modern musical instruments, the phrase *virtual polyphony* has been used to refer to the ability of an instrument to manipulate the harmonic series to create a spectral allusion of two interrelating musical lines (Bregman 1990; Wright & Bregman 1987). Expanding on Wright and Bregman’s (1987) observation of this phenomenon, perceiving sonic imagery as an interplay between intentionality and forefront material is a polyphonic sensory perception. Our capability for the simultaneous multi-layered perception of polyphonic sounds demonstrates that to understand sensory perception, acoustic spaces and sound experiences must be considered spectral (Warren 1999: 69). This polyphonic perception of space demonstrates the existence of a counterpoint between ambient and forefront or distinctively intentional noise which forms the basis of our subconscious and conscious sensitivity to sounds in space.

We hear in sound, and it is this immersive experience that must be emphasised in archaeoacoustic research. That the environments form a significant proportion of our perception of place and space is undeniable. A term that has developed concerning archaeacoustic studies into a landscape is *sound ecology* (Geisler 2014; Schafer 1977). Sound ecology is ‘the study of sounds in the landscape… [which] can be used to understand natural and human systems (Fuller et al. 2015: 207; Pijanowski et al. 2011). Navigating place via sound ecologies, through sonic perception, is significant, as is an investigation into place affecting sound-making (Primeau & Witt 2018: 875). As aforementioned, environmental factors play an important role in perceiving space and engaging with it. Uncovering a prehistoric soundscape means the communication component is seen as interlinked with that of the geophonic, biophonic, and anthrophonic (Blake & Cross 2015) rather than signalling, comprising the primary function of sound. Listening to the remote past requires this networked, spectral depiction of acoustics (Traux & Barrett 2011: 1204).

On the issue of expanding reconstructions of past sound, a different portion of the methodological approach in this study may be utilised for artistic reconstructions of past sound that, due to its surround sound capability, can be an immersive experience. With the results of the methodology, fieldwork, and acoustic analysis using MAX MSP 7, a prehistoric soundscape can be accessed through acoustic mapping that emphasises spatial dimensionality of sound.”

Psychoacoustic interpretations of the archaeoacoustic record have attempted to explore the connection between self and place (Primeau & Witt 2018: 875). However, this mode of interpretation remains speculative and ultimately internalises sensory experiences that persist beyond the parameters of the body and mind. Acoustic research should consider alternative visualisations of sound as a sensory experience. In doing so, sound ecologies and soundscapes of prehistory may be approached without the tendency to interpret and internalising experiential data as symbolic or sacred (Cox et al. 2020; Till 2019; Valenzuela et al. 2020; Waller 2015). Reducing the sonic past framed by visual stimuli, assemblages, and verbal language is illogical and inadequate. Sound is, after all, deeply integrated into all environments and thus dictates our experiences and reactions to it.

An essential aspect of approaching spatialised perception is a consideration of sonic depth. One approach is Goussios and Kitsion’s (2015) focus on reverberation. Reverberation combines resonance and vibrations embedded in layers of meaning and the physical perception of acoustics. They contemplate how reverberation ‘recreates existing places…[and] creates virtual spaces… [and for] narration’ within natural and built sonic environments (Goussios & Kitsion 2015: 4). By acknowledging that this reverberation is an ever-present feature of the environment, their definition suggests that reverberation dislocates interpretations of archaeological sites from materiality by instead emphasising resonance patterns in sound as the primary mode of analysis and thus, perception. Aside from prehistoric sites, contemporary practices in sound management (Hong & Jeon 2014; Vogiatzis & Remy 2017) take on board a broader perspective on acoustical awareness. Each environment, architectural or natural, carries its acoustic signature, culminating in its morphology size, and, if accurately discernible, relates with its purposes or uses by living beings.

By not containing the din of modernity, the prehistoric world would have presented a significantly quieter acoustic environment, where sound may have played a more significant role. Although the nature of our sonic world has evolved, the physical act of hearing has not. Although an obvious statement and in the face of visual bias in interpreting the archaeological record, ears have been a constant feature of hominid and human sensory perception. Although this methodology helps offer auditory context for the perception of certain sounds in space, methodological approaches should avoid limiting themselves to only testing between two points: sound material and the receptor. Restricting sensory perception to a series of dualities is problematic as it reduces the reality of experiencing a space sonically as a continuum. Peoples and cultures of the past were undoubtedly capable of sophisticated auditory perceptions of spaces and the decided manipulation and development of sound-making practices in reaction to acoustic landscapes.

Thinking of an acoustic environment as one with depth and volume is the function of *negative space*. It envisages the sonic experience of space without the dependency upon materiality to understand and analyse it. Instead, it acknowledges the necessity of materiality that may frame a space and its sonic qualities but does not limit our understanding or speculations of its function or meaning to materiality. The flexibility of the proposed *negative space* framework is that it emphasises the significance of visualising the depth of sound networks within internal spaces. Ultimately, listening is a three-dimensional, immersive, and continuous sensory experience. Beyond the notion of *negative space* offering a spatialised visualisation into the *depth* of acoustics, what becomes of the nature of listening and perceiving the breadth and complexity of the spectrum of sound?

In addition to polyphony relating to the movement of sound and its movement leading to our perception of acoustic depth within a space, repetition is necessary for sound behaviours. Similar to the metaphor mentioned earlier of silence as punctuation, repetition is required as a foundation for sound practices. Repetition in communication and signalling systems is necessary to make the repertoire of sound behaviour coherent. Sound is omnipresent, whether or not an ear is present to perceive it. For example, the generation of any pitch – something required for both language and musical intonation – is made possible through an accumulation of repeated sound periodicities (Margulis & Simchy-Gross 2016). A collection of ordered pitches can make a sentence or a melody. If silence is punctuation, repetition is necessary to establish the vocabulary in any sonic repertoire. To place this within archaeoacoustic methodologies, the notion of *negative space* and polyphonic perception can shift away from object-based analysis, with repetition or repeated behaviours helping to establish any evidence of intended sound-based behaviours.

### An Observational Case Study Applying Our Theoretical Propositions

An example of assessing the potential of polyphonic perception in a prehistorically inhabited space was carried out by Pham in 2018 at Coves del Toll. The karstic cave complex of Coves del Toll is situated north of Barcelona at the coordinates 41º 48’ 25” N and 2º 09’ 02” E, with the bulk of the archaeological seasons between 1952 and 1960. The cave systems have been interpreted as habitual dens for carnivores, with evidence of hominid habitation at two primary caves at the site (Bustos-Pérez 2017: 658). This includes Palaeolithic to Neolithic to Early Bronze Age occupation in Cova del Toll with La Cova de les Teixoneres that shows evidence for sporadic and seasonal Neanderthal occupation across the Middle Palaeolithic between 100 000BP to 40 000BP (Bustos-Pérez 2017: 658).

A basic overview of the occupation sequences at both caves is as follows. Cova del Toll, famous for having one of Europe’s most significant assemblages of prehistoric faunal remains, also has evidence of occupation ranging from the Epipalaeolithic to the Early Bronze Age. Cultural material located on site was diverse, ranging from stone tools – from projectile points to microliths – to bone tools to ceramics to burials (sourced courtesy of the Museu de Moià documentation in 2018, including notes from archaeologists Josep Maria Thomas and Francese Rovira). In contrast, La Cova de les Texioneres, which is located in the same Coves del Toll valley, holds evidence for periodic Neanderthal occupation from the Middle Palaeolithic (Sánchez-Hernández et al. 2014) with evidence for retouched tools in Unit III (Bustos-Pérez 2017). At both sites, it must be noted that there is considerable evidence for the overlapping of hominids and fauna, particularly carnivores, occupation at both caves (Blasco & Rosell 2009; Rufà et al. 2016).

To apply the notion of *negative space* to an archaeological site, the analysis of Coves del Toll (Figure I) focused on building an acoustic map identifying natural resonance points and mapping the acoustical features of a network of sound interactions between triangulated points that Pham determined throughout the caves. The two caves on the site that were analysed were primarily Cova del Toll and, as a secondary sonic exploration, La Cova de les Teixoneres. Due to widely different geological morphology, the occupied caves offered different sensory experiences. The occupation patterns at Cova del Toll were ideal for the extensive acoustic mapping at Coves dell Toll to curate a new form of acoustic technology where a structural visualisation of sound as a series of interacting networks can be trialled and analysed.

**Figure I:**
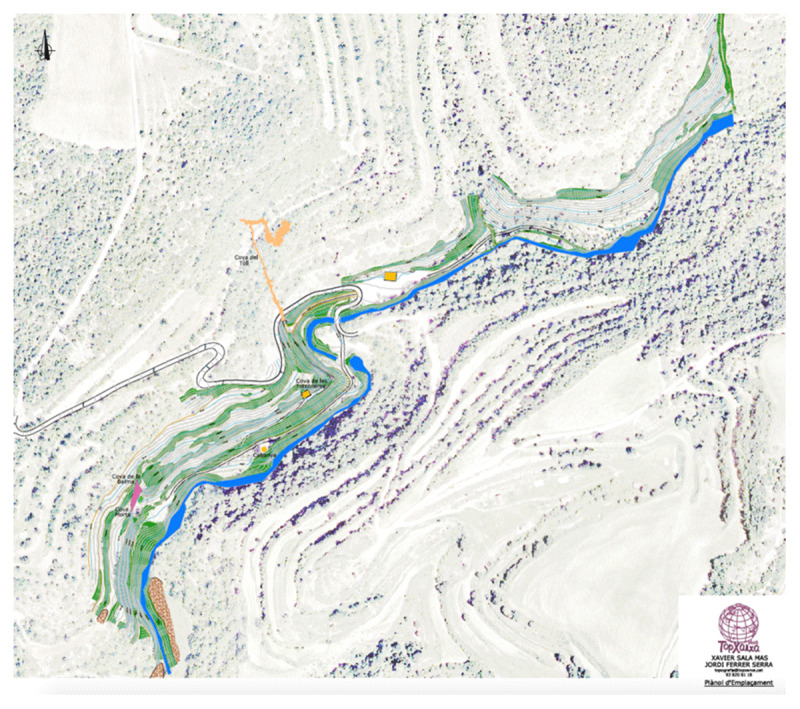
Map of the site of Coves del Toll (Image provided by Museu de Moià).

We conducted an observational survey on the external site before an interior acoustical study was completed on the two cave sites, Cova del Toll and La Cova de les Teixoneres. We proposed to develop a greater understanding of the sonic landscape through a series of ambient recordings. The inclusion of the ambience of the segments throughout both cave sites and complexes removed the reliance upon details concerning burial site locations or material culture that were documented in both areas. Those field recordings designed to capture the negative space of the sites were conducted in Cova del Toll and La Cova de les Teixoneres. Cova del Toll consists of two galleries, Galerie Sud (GS) and Galerie Est (GE), which were divided into manageable segments for recording and mapping (an example given in Figure II). The sections numbered per the maps the Museu de Moià provided, particularly regions with key assemblages and geological formations. Across both galleries, there was the attempt to, within possibilities with the geology of the karstic complex, have study sections maintain similar size or length. We selected the main chamber of La Cova de les Teixoneres (Figure III) was selected for acoustical testing because the smaller side sections were currently being utilised for storage of archaeological tools used by an ongoing season of excavations held by IPHES (Rosell et al. 2010a; Rosell et al. 2010b; Rufà et al. 2014; Sánchez-Hernández 2014). The sections labeled A, B, and C were segmented alongside IPHES labelling and geological shapes, like in Cova del Toll, to keep each mapped section to a similar length.

**Figure II:**
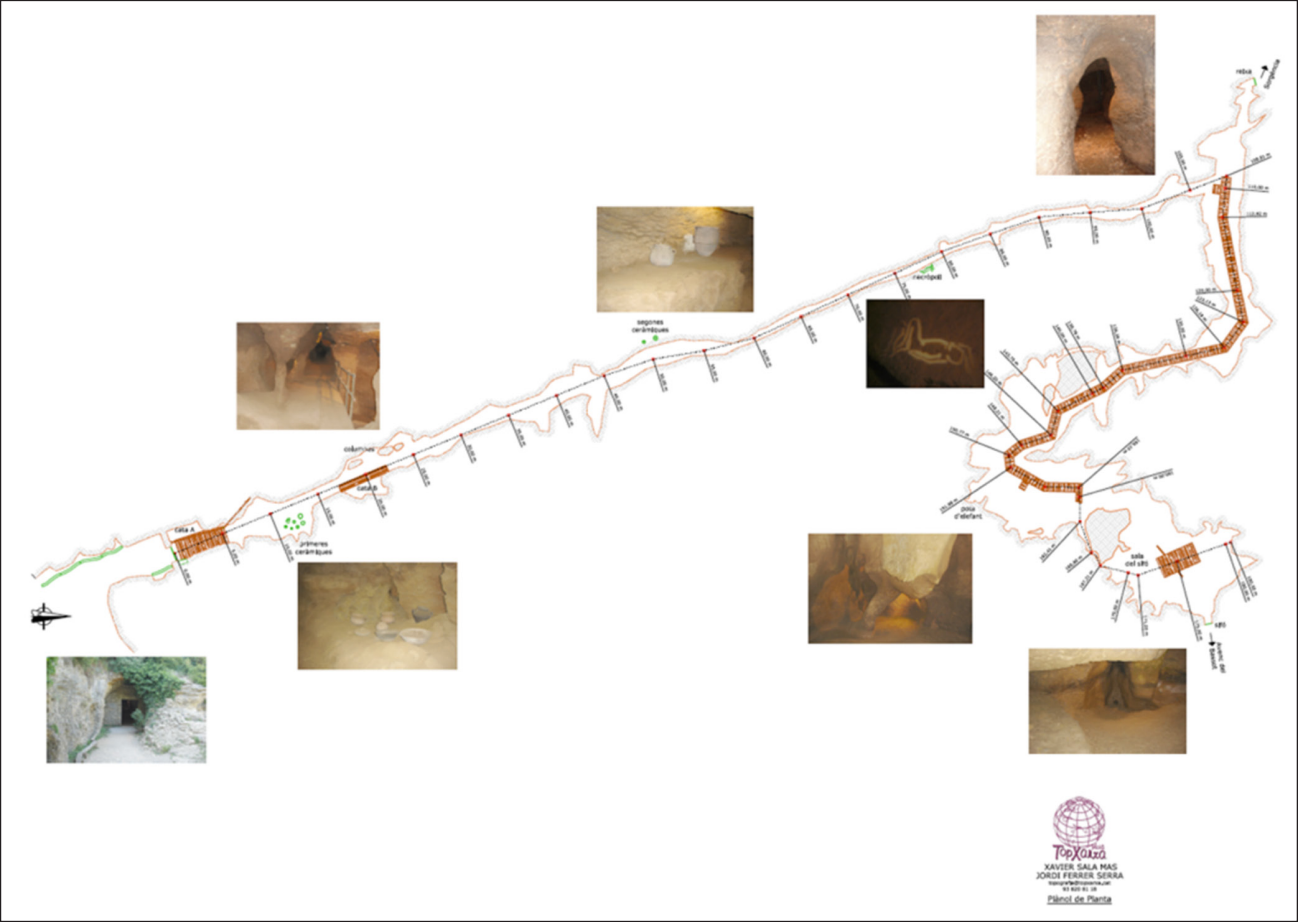
Cova del Toll gallery maps (Image provided by Museu de Moià). Galerie Sud (GS) and its features illustrated by the longer tunnel facing southward to the entrance. Galerie Est (GE) and its twisting geological features illustrated moving eastwards.

**Figure III:**
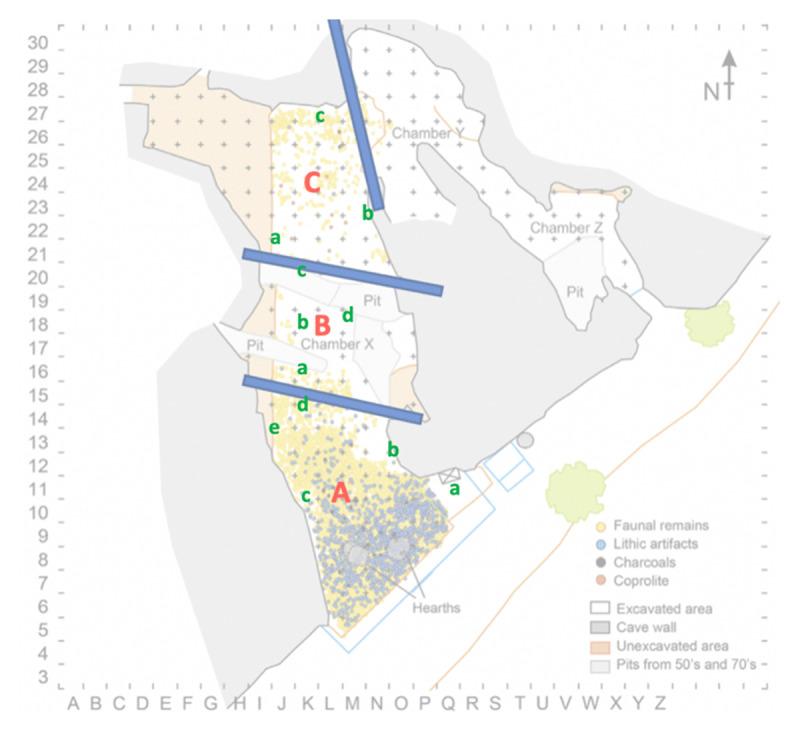
Each division per section of triangulated points of La Cova de les Teixoneres. Each section, (A, B, C) marked by bolded red capital letters, and each point of selected for triangulated recordings indicated by lower-case green letters. Original Diagram prior to divisions used for archaeoacoustic recordings is sourced from Rosell et al. 2010(a).

A selection of sound-producing objects and electronic sounds were used to produce sonic material for systematic recording (Table II) in the caves. The wide range of material was designed to test electronically generated and pre-recorded sounds, including three (low, mid, high) frequency tests, and live generated, including percussion and vocalisations (Table I). Instead of contemporary recordings that relied on locations of burial or materials excavated in prior archaeology seasons, all materials were chosen to test a range of sounds and their respective resonances throughout the space, with the electronically prepared signals used for impulse response testing. This was an experimental technique that recreated the materials for sound production that people of the remote past had access to on-site. These materials include stone, bone, wood, and body-generated sound. Here, it must be clarified that the objective of this study is the fixation on the location of materiality – i.e., rock art – framing the method of recording spaces where a correlation is made through the visual and the auditory (Table III). This also requires a degree of caution when the usual desire is to interpret such correlations through words or verbal-based meaning. Instead, the choice of including materials such as bone, in this instance, the knocking together of two fresh adult male cow femurs (one weighing 3.3 kg and the other 3.7 kg), stone, and wood were used to test and potentially demonstrate the diversity and timbral complexity of sounds that could be generated from the surrounding landscape and subsequently perceived by past people, not that these materials *mean* anything in symbolic or ritual terms (see Table IV for details).

**Table I:** Table categorising types of recording types.


TYPE	ABBREVIATION	MICROPHONE SET UP	NOTES

**Ambient**	AM	MS	External and Internal recording. Recordings taken in regions, sectors and way points.

**Resonant Central Point**	RCP	MS	Internal recording. Recordings taken at key marked points with notable artefact assemblages and burial.

**Triangulated Point**	TR	XY120^o^	Internal recording. Recordings cataloguing a range of sounds (pre-recorded and live) for the purposes of triangulated acoustic mapping.


**Table II:** Table categorising microphone setups used for field recordings, using a ZOOM H6N microphone for the recording process.


SET-UP TYPE	ABBREVIATION	TYPES OF RECORDINGS SET-UP USED FOR

**Mid-side Stereo**	MS	AM; RCP

**X-Y Pair Stereo**	XY 90^o^	TR

	XY 120^o^	TR


**Table III:** Range of sounds produced at each major cave. Extended List of sounds recorded and generated per point.


SITE	TYPES OF NOISES	

	ELECTRONICALLY PRE-RECORDED	LIVE GENERATED

**Cova del Toll** **GALERIE SUD**	LFS, LFL, MFS, MFL, HFS, HFL	WD, CL, BD, ST, BN, WH, SP, HA

**Cova del Toll** **GALERIE EST**	LFS, LFL, MFS, MFL, HFS, HFL	WD, CL, BD, ST, WH, SP, HA

**La Cova de les Texinores** **SECTORS A, B, C**	LFS, LFL, MFS, MFL, HFS, HFL	WD, CL, ST, BN, WH, SP, HA


Key for recording material: *Pre-recorded electronic sine tones* S – short L – Long LF – low frequency (500 Hz) MF – mid-frequency (5000 Hz) HF – high-frequency (19500 Hz) *Percussion material* WD – wood BD – body percussion CL – clapping ST – stone BN – bone *Vocalisations* WH – whisper SP – spoken HA – shouting

**Table IV:** **Triangulated Recordings Pre-recorded electronic sine tones**.


**(a) Cova del Toll: Galerie Sud** Triangulated mapping was conducted at select regions where the type of microphone setup was permitted by the geological features of the cave and in regions with primary evidence of human occupation and material.

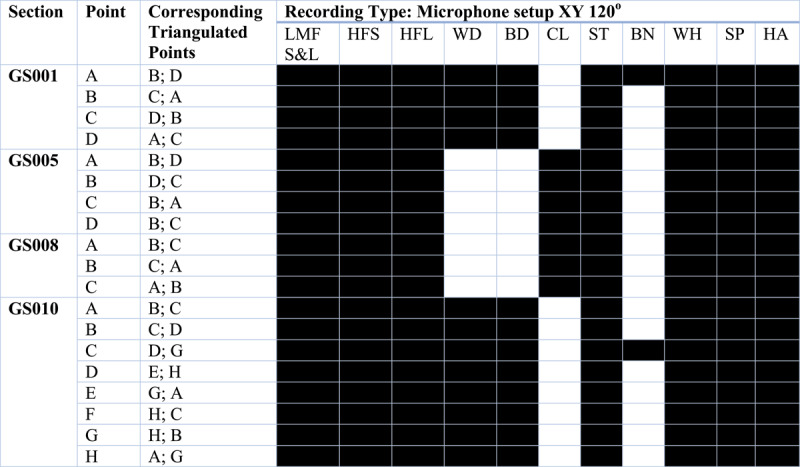

**Cova del Toll: Galerie Est** Only ambient recordings were taken in this gallery as there was no evidence for human occupation in this gallery. **(b) La Cova de les Teixoneres**

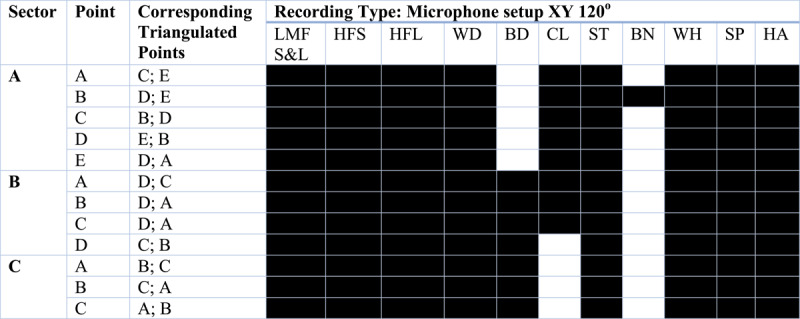


Key for recording material: **Hashccolored** squares indicate that recording type was successfully taken at that point *Pre-recorded electronic sine tones* S – short L – Long LF – low frequency (500 Hz) MF – mid-frequency (5000 Hz) HF – high-frequency (19500 Hz) *Percussion material* WD – wood BD – body percussion CL – clapping ST – stone BN – bone *Vocalisations* WH – whisper SP – spoken HA – shouting *Note on bone material used for recordings:* Adult Male Cow Femur 1, 3.2 kg, 34 cm × 17 cm × 12 cm Adult Male Cow Femur 2, 3.5 kg, 42 cm × 15 cm × 13 cm

Following this, each space was not recorded as a single signal, analysing movements between the sound source and the recording device. Instead, the chosen method was triangulating each section (Figure IV). This meant that points were taken around each segment between three and seven. Recordings were conducted in conjunction with two other points from each of these points, thus the term triangulation. Where possible, points were selected in sections where previous excavation reports had indicated key artefact assemblages or burials. This allowed for the contemporary ambience and acoustics of the karstic cave complex to be auralised and visualised, as well as creating a record of the resonance of the materials activated across each space. Ambient recordings and resonant central points were also taken to complete the sampled catalogue of sound sources produced from each triangulated and networked map (Table III and Table IV). As the material was eventually curated and analysed within studios with surround sound facilities, this provided a more accurate method to recreate or record the acoustic signature of a space or environment.

**Figure IV:**
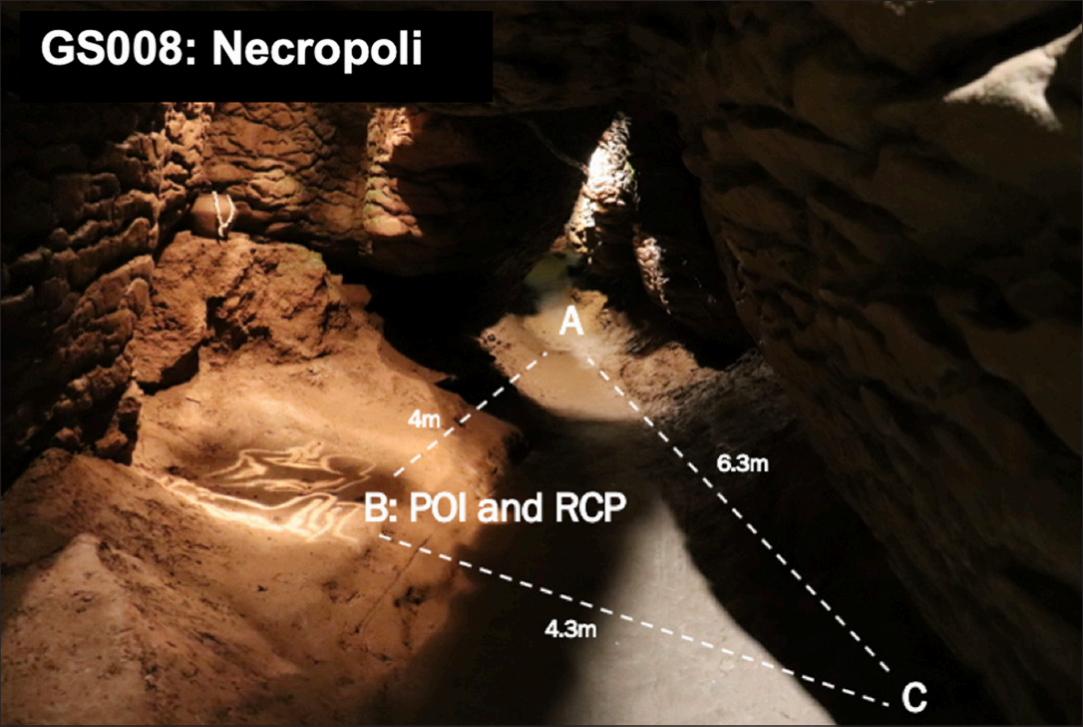
Sample of a division or segment of the cave complex and how the recordings were calculated. This figure shows the segment GS008 in Cova del Toll (also known as the Necropoli) being recorded from points A, B and C. RCP refers to the Resonant Central Point which was marked to capture the ambient noise with a M-S microphone set up, whereas each of the triangulated points (POI recordings at A, B and C) were collected using a XY-120o microphone set up. For more details referring to the forms of sounds collected and materials used to sample sounds, refer to the tables provided.

The results in the resonance data recorded no notable changes in resonance or reverberations in the regions that previously recorded hominin activity. In particular, this finding pertains to any locations that may be interpreted as sacred, such as the location of material culture throughout both sites or the burials documented in Section A in La Cova de les Teixoneres or GS008 in Cova del Toll (Figure IV). Instead, capturing each space’s ambient acoustics alongside various locally sourced material activations, the acoustic data taken from Coves del Toll created an accurate aural map of the site in its contemporary condition. Rather than framing the acoustics as a directed influence on behaviour, this three-dimensional and spatialised capturing of acoustics at Cove del Toll can demonstrate a holistic presentation of the sonics of the cave system, which can be applied to understanding the general sonic foundation upon which hominin habitation and activity occurred. Furthermore, this data permits an acoustic depiction of the UNESCO heritage site that can be applied for sensory conservation purposes.

As a preliminary insight into how the theoretical proposal of *negative space* may be applied to a prehistoric archaeological site, the approach combined the speculative elements that experimental archaeology may utilise. This included recording local materials to replicate the sonic possibilities across the Epipalaeolithic to Early Bronze Age occupation (Sánchez-Hernández et al. 2014), with impulse response testing with electronic signals. The combination permitted a networked approach, combining ambient recordings of the karstic complex spaces and their resonances with experimental archaeological concepts. As we listen holistically, archaeoacoustics informed by these principles shift from a study of resonant points to spatial resonance. As Primeau and Witt (2018) note of the burgeoning phenomenological approach to acoustic landscapes, ‘this experience of navigating space creates place.’ We may not be able to hear the past truly, but it may be possible by detaching sound from materiality and verbal meaning to focus on how it can model the sonic experience and acoustic landscapes of the remote past.

## Discussion: Listening backwards

Within archaeoacoustical research, we propose that the general issue is making tactile and visual invisible sensations to structure our understanding of sound. Relying on physical traces of the past remains inevitable in an archaeological investigation; however, a systematic move in acoustic research to understand the nature of sound in space stems not from materiality but from its operational qualities of sound.

The collective imagination of our past can begin to evolve beyond the seemingly silent. This focus in archaeoacoustics streamlines the intentionality component of sound as interpreted by word, whereas sound is fundamentally non-verbal and spectral. To question the purpose of sound is to enforce the notion that all sound must be coded or culturally symbolic. This fixation with the *function* of sound is to forgo its ubiquity. An evolving theoretical framework that emphasises spatial depth and sound as a spectral experience is essential to listen backward. Archaeoacoustic research can begin to investigate both the transformation of sound spaces and the nature of cognitive evolution concerning sensory perception and hominid relationships with the physical world.

This paper’s theoretical assessment of archaeoacoustics focuses on the complexity of sonic perception and its relationship to sociality and the environment. This archaeoacoustic study focuses on the systematic shift toward a structural perspective of sound. The proposed theoretical framework is founded upon visualising sites’ sounds as *negative space*. This is not to say that spaces are sonically empty but to suggest a terminological shift towards an analytical interpretation of spaces used by humans as ones that carry the interpretation of resonance throughout spatial and temporal extents of space and sound to conceptualise depth.

As current paradigms of acoustic research highlight the requirement for identifying intentionality, a spectralist approach to sound brings to the fore the significance of “silence” and ambient noise. To offer an example of how our proposal can be applied to experimental archaeological consideration of acoustics, we showed how a synthesis of experimental techniques, such as the sound recording of various materials with an ambient and resonance assessment of an interior site, can be applied. The site of Coves del Toll provided a site in which the testing of archaeological theories that tied material finds, or burial sites with potential acoustic meaning was conducted. The site resulted in no particular contemporary resonances aligning with historical notes of excavation finds or burial sites at Coves del Toll while allowing for an observational study of the nature of karstic cave acoustics. The spatial depth of *negative space* is reinforced through a similar depth in auditory imagery. This necessity for three-dimensional visualisation of sound then calls into question the notion of experience and perception. Sound is not felt or seen in the sense that an archaeologist might uncover an artefact but is inescapably heard.

The concepts of spectralism and polyphony demonstrate how the manipulation of sound within communication systems can extend beyond sound-producing objects or the potential evidence for prehistoric music-making. It remains necessary to reiterate that the reference to music-making within archaeoacoustic research is not intended to relate to a contemporary music mindset. Contemporary music is often dominated by text or imagined as orchestral, but it should be viewed as a communicative system when considering past musical developments, mainly percussion, and vocalisation. As music does not rely on verbal meaning and can transmit multiple streams of information, our ability to perceive the complexity of music in a single image suggests that it potentially holds deep ancestry as a communication system within hominid societies. The theoretical model proposed by this essay is designed to combine principles of music, the concept of *negative space*, and the spectral reality of sound in order to broaden the application of archaeoacoustical methodologies and analysis to a broader range of spaces and structures through time and to the evolution of human sonic behaviors.

## Data Accessibility Statement

This study did not involve human remains or materials.
